# 2% chlorhexidine gluconate aqueous versus 2% chlorhexidine gluconate in 70% isopropyl alcohol for skin disinfection prior to percutaneous central venous catheterisation: the ARCTIC randomised controlled feasibility trial

**DOI:** 10.1136/archdischild-2023-325871

**Published:** 2023-10-31

**Authors:** Paul Clarke, Aung Soe, Amy Nichols, Helen Harizaj, Mark A Webber, Louise Linsell, Jennifer L Bell, Catherine Tremlett, Priyadarsini Muthukumar, Santosh Pattnayak, Christopher Partlett, Andrew King, Ed Juszczak, Paul T Heath

**Affiliations:** 1 Neonatal Intensive Care Unit, Norfolk and Norwich University Hospitals NHS Foundation Trust, Norwich, Norfolk, UK; 2 Norwich Medical School, University of East Anglia, Norwich, Norfolk, UK; 3 Neonatal Intensive Care Unit, Medway Maritime Hospital, Gillingham, Kent, UK; 4 Quadram Institute Bioscience, Norwich, Norfolk, UK; 5 National Perinatal Epidemiology Unit, Nuffield Department of Population Health, University of Oxford, Oxford, UK; 6 Department of Microbiology, Norfolk and Norwich University Hospitals NHS Foundation Trust, Norwich, Norfolk, UK; 7 Centre for Neonatal and Paediatric Infection, Infection and Immunity, Saint George's University of London, London, UK

**Keywords:** Infectious Disease Medicine, Microbiology, Neonatology, Sepsis

## Abstract

**Objective:**

Catheter-related sepsis (CRS) is a major complication with significant morbidity and mortality. Evidence is lacking regarding the most appropriate antiseptic for skin disinfection before percutaneous central venous catheter (PCVC) insertion in preterm neonates. To inform the feasibility and design of a definitive randomised controlled trial (RCT) of two antiseptic formulations, we conducted the Antiseptic Randomised Controlled Trial for Insertion of Catheters (ARCTIC) feasibility study to assess catheter colonisation, sepsis, and skin morbidity.

**Design:**

Feasibility RCT.

**Setting:**

Two UK tertiary-level neonatal intensive care units.

**Patients:**

Preterm infants born <34 weeks’ gestation scheduled to undergo PCVC insertion.

**Interventions:**

Skin disinfection with either 2% chlorhexidine gluconate (CHG)-aqueous or 2% CHG-70% isopropyl alcohol (IPA) before PCVC insertion and at removal.

**Primary outcome:**

Proportion in the 2% CHG-70% IPA arm with a colonised catheter at removal.

**Main feasibility outcomes:**

Rates of: (1) CRS, catheter-associated sepsis (CAS), and CRS/CAS per 1,000 PCVC days; (2) recruitment and retention; (3) data completeness.

**Safety outcomes:**

Daily skin morbidity scores recorded from catheter insertion until 48 hours post-removal.

**Results:**

116 babies were randomised. Primary outcome incidence was 4.1% (95% confidence interval: 0.9% to 11.5%). Overall catheter colonisation rate was 5.2% (5/97); CRS 2.3/1000 catheter days; CAS 14.8/1000 catheter days. Recruitment, retention and data completeness were good. No major antiseptic-related skin injury was reported.

**Conclusions:**

A definitive comparative efficacy trial is feasible, but the very low catheter colonisation rate would make a large-scale RCT challenging due to the very large sample size required. ARCTIC provides preliminary reassurance supporting potential safe use of 2% CHG-70% IPA and 2% CHG-aqueous in preterm neonates.

**Trial registration number:**

ISRCTN82571474.

**WHAT IS ALREADY KNOWN ON THIS TOPIC**

- Good skin disinfection is vital prior to central venous catheterisation to minimise risk of catheter colonisation and subsequent sepsis.
- The skin of preterm neonates is particularly vulnerable to antiseptic chemical burn injury.
- The most effective antiseptic for reducing risks of both catheter sepsis and skin harms in preterm neonates is unknown due to lacking clinical trial evidence.

**WHAT THIS STUDY ADDS**

- The ARCTIC study provides contemporary evidence for rates of catheter-related infections associated with pre-procedural skin disinfection using topical 2% CHG-70% IPA and 2% CHG-aqueous solutions.
- Use of 2% CHG-70% IPA for central venous catheterisation in preterm neonates is associated with a very low rate of catheter colonisation at catheter removal.
- The robust safety data obtained would support the use of these agents in a large comparative trial, with skin application adhering to strict guidelines.

**HOW THIS STUDY MIGHT AFFECT RESEARCH, PRACTICE OR POLICY**

The ARCTIC study results provide an accurate indication of the very large sample size that would be needed for a definitive comparative non-inferiority antiseptic trial.

## Introduction

Percutaneous central venous catheters (PCVCs) are essential, but pose a significant risk for bloodstream infection.^1–3^ Catheter-related and catheter-associated sepsis (CRS and CAS) are dangerous complications that carry significant neonatal morbidity. Sepsis increases intensive care days, antibiotic usage, and risk of adverse neurodevelopmental outcomes and death.^4–7^

Reducing CRS remains a major goal of the NHS.^8^ Adoption of catheter care ‘bundles’ helps reduce CRS rates,^9 10^ but with a multifactorial aetiology the goal of zero CRS still proves elusive.^11 12^ Individual components of bundles have rarely been rigorously studied through randomised controlled trials (RCTs) in neonates.^3 10 12^ One crucial component in preventing catheter infection is optimal antiseptic choice for pre-procedural skin disinfection of the catheter insertion site.^2 13^ Studies in adults, including meta-analysis, show that alcohol-based antiseptics are superior for topical antisepsis.^14 15^ UK evidence-based guidelines in adults and older children recommend 2% chlorhexidine gluconate (CHG) in 70% isopropyl alcohol (2% CHG-70% IPA),^16 17^ but they lack guidance on preferred antiseptic in preterm infants, reflecting the paucity of evidence and safety concerns specific to this population.^3 13 18^ Consequently, multiple different antiseptics, concentrations and combinations are in use in UK neonatal intensive care units (NICUs).^19 20^

No published RCT has so far examined the safety and efficacy of alcohol-based versus aqueous CHG formulations for skin antisepsis prior to PCVC insertion in preterm neonates. We therefore undertook the Antiseptic Randomised Controlled Trial for Insertion of Catheters (ARCTIC) feasibility study to inform the safety, design and scale of a potential large-scale multicentre RCT to determine whether 2% CHG-aqueous is non-inferior in antiseptic efficacy compared with 2% CHG-70% IPA for skin disinfection prior to PCVC insertion.

## Methods

### Study design and setting

A blinded parallel group feasibility RCT conducted in two UK tertiary-level NICUs: Norfolk and Norwich University Hospital, and Medway Maritime Hospital.

### Participants

Preterm infants born at <34 weeks’ gestation were eligible if they required PCVC insertion for parenteral nutrition. We excluded infants: unlikely to survive; with a life-threatening congenital abnormality or an underlying skin condition; who already had an indwelling PCVC or were previously enrolled; with a new episode of suspected sepsis with commencement of antibiotics within the previous 48 hours; with a positive blood culture (BC) within the previous 7 days without a subsequent negative culture.^3^

### Antiseptic products and blinding

The two topical Investigational Medicinal Product (IMP) antiseptic agents used, 2% CHG-aqueous and 2% CHG-70% IPA, were specially manufactured under licence for this trial. Production, labelling and blinding of study packs containing paired bottles each containing 20 mL of IMP was as described.^3^

### Randomisation

Secure internet-based randomisation was performed as close to catheter insertion as possible by a research team member or trained clinician.^3^ The randomisation system used stratified block randomisation with allocation sequence generated by the senior trials statistician (LL). Blocks of size 4 and 8 were generated using Stata (V.13/SE for Windows). Stratification was by centre and gestational age at birth (<28 weeks and 28^+0^ to 33^+6^ weeks). Allocation was weighted 3:1 in favour of the 2% CHG-70% IPA IMP group to inform the primary objective of sample size calculation for a phase-III trial.^3^

### Interventions

The trial procedures have been published in detail.^3^ Trained clinical staff inserted and removed PCVCs according to the trial’s protocol and working good clinical practice guidelines for catheter insertion and removal (online supplemental files 1 and 2). Specimens collected on catheter removal were: (1) two exit site skin swabs (ESSSs), one before and one after skin disinfection of insertion site using the same allocated IMP as at catheter insertion; (2) two ~1 cm long catheter segments, namely the tip plus a proximal segment taken approximately 1-2 cm distal to the former skin entry point; and (3) a peripheral BC (only if catheter removal was for suspected sepsis).^3^

10.1136/archdischild-2023-325871.supp1 Supplementary data

10.1136/archdischild-2023-325871.supp2 Supplementary data

### Catheter-related sepsis, catheter colonisation and catheter-associated sepsis

Our study had strict definitions for definite CRS, catheter colonisation and CAS (table 1, footnotes).

**Table 1:** Summary efficacy outcomes for bacteriology and sepsis including primary outcome

	2% CHG-70% IPA (n=79)	2% CHG-aqueous (n=27)	All (n=106)
Positive exit site skin swab at catheter removal before disinfection, n (%)	11 (15.1)	4 (16.7)	15 (15.5)
Missing	6	3	9
Positive exit site skin swab at catheter removal after disinfection, n (%)	1 (1.4)	1 (4.3)	2 (2.1)
Missing	7	4	11
Culture-positive catheter segment at removal†, n (%)	3 (4.1)*	2 (8.3)	5 (5.2)
Positive tip alone	1 (1.3)	1 (3.7)	2 (1.9)
Positive proximal segment alone	2 (2.5)	0	2 (1.9)
Both tip and proximal segment positive	0	1 (4.2)	1 (1.0)
Missing	6	3	9
Definite catheter-related sepsis‡, n (%)	1 (1.5)	1 (4.5)	2 (2.3)
Missing	13	5	18
Catheter-associated sepsis§, n (%)	10 (13.7)	3 (12.5)	13 (13.4)
Missing	6	3	9
Total number of PCVC days	653	223	876
Definite catheter-related sepsis, n (rate per 1000 PCVC days)	1 (1.5)	1 (4.5)	2 (2.3)
Catheter-associated sepsis, n (rate per 1000 PCVC days)	10 (15.3)	3 (13.5)	13 (14.8)

*Primary outcome: 3/73 (4.1%) with 95% confidence interval of 0.9% to 11.5%.

†Catheter colonisation: a catheter that at the time of removal has either one or both segments culture positive.

‡Definite catheter-related sepsis: a peripheral BC plus any catheter segment (i.e. proximal and/or tip) positive with the same organism, based on bacterial culture, antibiotic sensitivity and molecular typing, from a neonate who had an indwelling PCVC and clinical signs of sepsis but no other focus of sepsis.

§Catheter-associated sepsis: clinical signs of sepsis and an accompanying positive BC in the period between catheter insertion and 48 hours post removal but with no other focus of sepsis and with both catheter segment cultures negative.

BC, blood culture; CHG, chlorhexidine gluconate; PCVC, percutaneous central venous catheter.

### Microbiological and molecular analysis

Catheter segments, skin swabs, and BCs underwent routine culture and antibiotic sensitivities in our hospital microbiology laboratories. Bacterial growths from ESSS cultures were assessed semi-quantitatively.^21^ Culture-positive isolates were retained for whole genome sequencing, allowing for unequivocal diagnosis of CRS.^3^

### Outcome measures and assessments

#### Primary outcome

Proportion of babies in the 2% CHG-70% IPA group with catheter colonisation, determined by at least one of the two catheter segments taken at catheter removal being bacterial culture positive.

#### Secondary outcomes

##### Efficacy outcomes

(1) Proportion of infants with positive ESSSs (pre disinfection and post disinfection) at catheter removal; (2) number and type of culture-positive catheter segments at removal; (3) bacterial species identified on positive BC, ESSSs and catheter segments as typed by molecular methods (undertaken to prove concordance of paired blood and catheter isolates to a species level for definitive diagnosis of definite CRS); (4) proportion of infants with definite CRS in the period between catheter insertion and 48 hours post catheter removal; (5) proportion of infants with CAS in the period between catheter insertion and 48 hours post catheter removal; (6) rate of CRS per 1000 PCVC days; (7) rate of CAS per 1000 PCVC days; (8) rates of recruitment and retention; (9) views of parents and clinicians on factors affecting recruitment and retention; (10) proportion of infants completing the study with complete data for the primary outcome; and (11) proportions of infants with missing data collection forms.

##### Safety outcomes

Skin condition and morbidity, assessed at catheter insertion and daily until 48 hours post catheter removal. A validated neonatal contact dermatitis scoring system was used,^22^ with minor modification.^3^

##### Process outcomes

(1) Adherence to study protocol; (2) numbers of attempted and failed catheterisations; and (3) withdrawals.

### Sample size and statistical analysis

A target sample size comprising ~93 babies having successfully inserted catheters would suffice to estimate the anticipated incidence of the primary outcome (20%) in the reference 2% CHG-70% IPA group with a 95% CI of 11% to 31%.^3^ A statistical analysis plan was developed and approved by the Trial Steering Committee (TSC) chair by the end of enrolment (online supplemental file 3). This feasibility study is reported in accordance with the Consolidated Standards of Reporting Trials extension guidelines for randomised pilot and feasibility trials.^23^

10.1136/archdischild-2023-325871.supp3 Supplementary data

### Data management

Outcome data were collected as described,^3^ using study-specific forms. Data were transferred and stored in compliance with Good Clinical Practice (GCP) and Data Protection legislation.^3^

### Monitoring

The Sponsor’s nominated representatives undertook regular monitoring visits during the course of the trial, according to a monitoring plan.^3^

### Pharmacovigilance, data and safety monitoring

Pharmacovigilance was conducted as described.^3^ The trial had a Data Monitoring Committee (DMC) and TSC with respective charters signed off by their independent chairs prior to first enrolment. The DMC met regularly before, during and at the end of the trial to review the protocol, compliance, safety and outcome data, including after the first 50 babies were enrolled.^3^

### Patient and public involvement

The study was developed with extensive parent and public input.^3^ Two lay TSC parent members assisted dissemination of a final summary report to parents of all participants.

### Ethics and regulatory approvals

A clinical trial authorisation was granted by the responsible authority on 23rd October 2015 (MHRA reference: 13630/0009/001-0001).

## Results

**Figure 1:**
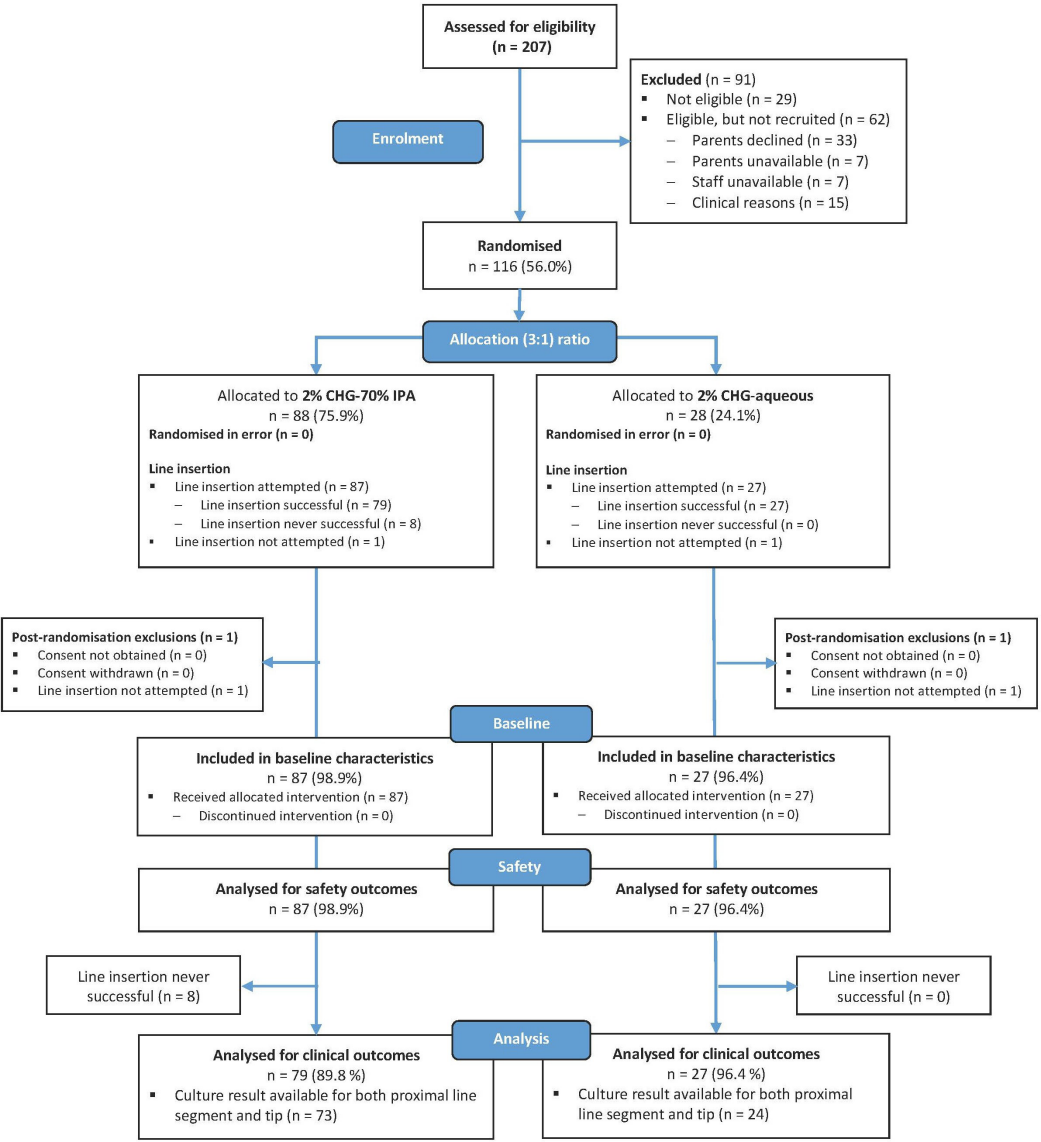
Study flow diagram.

Between March 2017 and July 2018, 207 infants were assessed for eligibility. 116 were randomised of whom 88 were allocated 2% CHG-70% IPA and 28 were allocated 2% CHG-aqueous (figure 1). Table 2 presents baseline characteristics of all 114 babies who underwent attempted catheterisation. Additional details relating to catheterisation are provided (online supplemental table S1).

10.1136/archdischild-2023-325871.supp4 Supplementary data

**Table 2:** Infant and maternal baseline characteristics

	2% CHG-70% IPA (n=87)	2% CHG-aqueous (n=27)
Centre*, n (%)		
Norfolk and Norwich	56 (64.4)	17 (63.0)
Medway	31 (35.6)	10 (37.0)
Male sex, n (%)	46 (52.9)	13 (48.1)
Infant’s birth weight (g)		
Mean (SD)	1089 (340.5)	1075 (366.3)
Range	(508–2150)	(575–1900)
<500 g	0	0
500 to 999 g	39 (44.8)	15 (55.6)
1000 to 1499 g	37 (42.5)	8 (29.6)
≥1500 g	11 (12.6)	4 (14.8)
Gestational age at birth* (completed weeks)		
Median (IQR)	28 (26–30)	28 (26–30)
Range	(23–32)	(23–33)
<26^+0^	20 (23.0)	5 (18.5)
26^+0^ to 27^+6^	19 (21.8)	7 (25.9)
28^+0^ to 33^+6^	48 (55.2)	15 (55.6)
One of a multiple pregnancy, n (%)	16 (18.4)	9 (33.3)
Mode of delivery, n (%)		
Vaginal	29 (33.3)	7 (25.9)
Caesarean	58 (66.7)	20 (74.1)
Membranes ruptured prior to labour, n (%)	35 (41.7)	9 (36.0)
>24 hours before delivery	20 (57.1)	6 (66.7)
≤24 hours before delivery	15 (42.9)	3 (33.3)
Unknown	0	0
Apgar score at 5 minutes		
Median (IQR)	8 (7–9)	8 (6–9)
<4	2 (2.4)	2 (7.7)
First recorded temperature on admission to NICU after birth, mean (SD) °C	36.8 (0.7)	36.8 (0.9)
Infant ventilated via an endotracheal tube at the time of randomisation, n (%)	34 (39.1)	13 (50.0)
Infant born in recruiting hospital, n (%)	68 (78.2)	19 (70.4)
Any surgical procedure prior to randomisation, n (%)	6 (6.9)	1 (3.7)
Prophylactic antifungal medication at the time of randomisation, n (%)	27 (31.0)	9 (33.3)
Received antibiotics prior to randomisation†, n (%)	78 (98.7)	26 (96.3)
Devices in situ at time of PCVC insertion†, n (%)		
Chest drain	1 (1.3)	0 (0.0)
Endotracheal tube	28 (35.4)	12 (44.4)
Peripheral arterial line	2 (2.5)	2 (7.4)
Peripheral venous cannula	70 (88.6)	18 (66.7)
Umbilical arterial catheter	24 (30.4)	11 (40.7)
Uumbilical venous catheter	43 (54.4)	14 (51.9)
Other	2 (2.5)	0 (0.0)
Mother’s age (years), mean (SD)	29.7 (6.0)	29.4 (5.7)
Received any antenatal corticosteroids, n (%)	80 (92.0)	24 (88.9)
Received antibiotics within the week before delivery, n (%)	27 (31.0)	8 (29.6)
Feverish in labour (temperature>38.0°C)‡, n (%)	4 (4.8)	0 (0.0)
Chorioamnionitis suspected clinically before delivery, n (%)	7 (8.0)	1 (3.7)

Unless otherwise stated, data are n (%). SD, standard deviation; IQR, interquartile range.

*Stratification factors.

†Data missing for eight cases in the 2% CHG-70% IPA group.

‡Data missing for three cases in the 2% CHG-70% IPA group.

CHG, chlorhexidine gluconate; IPA, isopropyl alcohol; NICU, neonatal intensive care unit; PCVC, percutaneous central venous catheter.

### Efficacy outcomes

#### Clinical and microbiological outcomes including primary outcome

One hundred and six babies were assessed for clinical and microbiological outcomes (figure 1). Table 3 shows individual results for the 31 babies who had at least one positive culture result isolated from culture of blood, ESSSs and catheter segments. Paired catheter segment culture results were available for 97 babies, losses mainly being due to repatriation of neonates to non-participating hospitals before catheter removal. The overall catheter colonisation rate was 5.2% (5/97). Of 79 babies allocated the 2% CHG-70% IPA antiseptic and successfully catheterised, 73 had paired catheter segments available and 3 babies had a colonised catheter at the time of removal, an incidence for the primary outcome of 4.1% (95% CI 0.9% to 11.5%). One baby in each group had definite CRS (2% CHG-70% IPA 1.5% (1/66) vs 2% CHG-aqueous 4.5% (1/22)), and rates of CAS were similar (2% CHG-70% IPA 13.7% vs 2% CHG-aqueous 12.5%). The bacteriology and sepsis-related secondary outcomes are summarised by allocation in table 1. At catheter removal, 15 babies (15.5%) overall had a culture-positive ESSS pre-disinfection, with proportions similar between groups, and only one baby in each group had a positive ESSS post-disinfection (tables 1 and 3).

**Table 3:** Bacterial species isolated via standard microbiology laboratory culture for infants with at least one positive culture result

ID no	IMP allocation	Blood culture(s)				Exit site skin swab		Catheter segment	
		Closest to PCVC removal (days)	Blood culture results			Before disinfection	After disinfection	Proximal	Tip
			#1	#2	#3				
1	2% CHG-70% IPA	6.2 pre	No growth	No growth	–	CoNS: *S. capitis*	No growth	No growth	No growth
2	2% CHG-70% IPA	–	N/A	N/A	N/A	No growth	No growth	CoNS: *S. capitis*	No growth
3	2% CHG-70% IPA	0.3 pre	Mixed CoNS (not specified)	No growth	–	No growth	No growth	No growth	No growth
4	2% CHG-70% IPA	–	N/A	N/A	N/A	CoNS: *S. haemolyticus*	No growth	No growth	No growth
5	2% CHG-70% IPA	0.0 post	CoNS: *S. epidermidis*	CoNS: *S. capitis*	–	No growth	No growth	No growth	No growth
6	2% CHG-70% IPA	1.8 post	No growth	–	–	CoNS: *S. capitis*	No growth	No growth	No growth
7	2% CHG-70% IPA	0.9 post	No growth	–	–	No growth	CoNS: *S. capitis*	No growth	No growth
8	2% CHG-70% IPA	–	N/A	N/A	N/A	CoNS: *S. epidermidis*	No growth	No growth	No growth
9	2% CHG-70% IPA	–	N/A	N/A	N/A	Mixed CoNS (not specified)	No growth	No growth	No growth
10	2% CHG-70% IPA	1.3 post	CoNS: (not specified)	–	–	No growth	No growth	No growth	No growth
11	2% CHG-70% IPA	0.0 post	No growth	CoNS: *S. capitis*	–	No growth	No growth	No growth	No growth
12	2% CHG-70% IPA	–	N/A	N/A	N/A	CoNS: *S. capitis*	No growth	No growth	CoNS: *S. capitis*
13	2% CHG-70% IPA	1.4 post	No growth	CoNS: *S. haemolyticus*	–	No growth	No growth	No growth	No growth
14*	2% CHG-70% IPA	0.0 post	CoNS: 1. *S. haemolyticus*; 2. *S. epidermidis*	No growth	–	CoNS: *S. capitis*	No growth	No growth	No growth
15†	2% CHG-70% IPA	1.7 post	CoNS: *S. capitis*	CoNS: *S. capitis*	No growth	No growth	No growth	CoNS: *S. capitis*	No growth
16*	2% CHG-70% IPA	6.1 pre	No growth	CoNS: *S. capitis*	–	CoNS: *S. capitis*	No growth	No growth	No growth
17	2% CHG-70% IPA	0.2 pre	CoNS: *S. haemolyticus*	No growth	CoNS: *S. capitis*	No growth	No growth	No growth	No growth
18	2% CHG-70% IPA	5.8 pre	CoNS: *S. haemolyticus*	*CoNS: S. epidermidis*	No growth	No growth	No growth	No growth	No growth
19	2% CHG-70% IPA	–	N/A	N/A	N/A	CoNS: *S. warneri*	No growth	No growth	No growth
20	2% CHG-70% IPA	0.2 pre	No growth	CoNS: *S. capitis*	No growth	No growth	No growth	No growth	No growth
21	2% CHG-70% IPA	–	N/A	N/A	N/A	1. CoNS: *S. capitis*; 2. *S*. *aureus*	No growth	No growth	No growth
22	2% CHG-70% IPA	–	CoNS: not specified	–	–	Missing‡	Missing‡	Missing‡	No growth
23	2% CHG-70% IPA	–	N/A	N/A	N/A	CoNS: *S. haemolyticus*	No growth	No growth	No growth
24	2% CHG-aqueous	1.7 post	No growth	–	–	CoNS: *S. haemolyticus*	No growth	No growth	No growth
25	2% CHG-aqueous	0.0 post	CoNS: *S. warneri*	No growth	–	No growth	No growth	No growth	No growth
26†	2% CHG-aqueous	0.3 pre	No growth	CoNS: *S. warneri*	–	No growth	No growth	No growth	CoNS: *S. warneri*
27	2% CHG-aqueous	–	N/A	N/A	N/A	CoNS: *S. haemolyticus§*	No growth	No growth	No growth
28	2% CHG-aqueous	–	N/A	N/A	N/A	CoNS: *S. capitis*	CoNS: *S. capitis*	No growth	No growth
29	2% CHG-aqueous	4.0 pre	CoNS: *S. capitis*	No growth	–	No growth	No growth	No growth	No growth
30	2% CHG-aqueous	0.5 post	CoNS: 1. *S. epidermidis*; 2. *S. capitis*	No growth	–	No growth	Missing¶	No growth	No growth
31	2% CHG-aqueous	–	N/A	N/A	N/A	CoNS: *S. epidermidis*	No growth	CoNS: *S. epidermidis*	CoNS: *S. epidermidis*

*One of two cases of catheter-associated sepsis.

†One of two cases of definite catheter-related sepsis, both paired isolates confirmed via whole genome sequencing.

‡Infant was transferred to a non-participating site where their line was removed.

§Detail of species was not captured in database, but was found post data lock.

¶Sample not obtained.

CoNS, coagulase-negative staphylococcus; ID, identifier; IMP, Investigational Medicinal Product; N/A, not applicable because no blood culture taken between catheter insertion and 48 hours post removal; PCVC, percutaneous central venous catheter; *S. aureus*, *Staphylococcus aureus*; *S. capitas*, *Staphylococcus capitis*; *S. epidermidis*, *Staphylococcus epidermidis*; *S. haemolyticus*, *Staphylococcus haemolyticus*; *S. warneri*, *Staphylococcus warneri*.

Paired bacterial isolates from relevant babies underwent whole genome sequencing for definitive speciation. Specimens of particular interest were blood and catheter isolates in the two CRS cases (ID numbers 15 and 26, table 3), and the blood and ESSS isolates in the two CAS cases (ID numbers 14 and 16, table 3). Genome sequencing confirmed identity and exact match of the CoNS species in both the CRS cases. Unfortunately, the paired BC isolates were not retained for the two CAS cases, so their typing and matching was not possible.

#### Recruitment, retention and factors affecting

Of 178 eligible infants, we approached the parents of 149 and 116 (77.9%) gave consent. The overall retention rate was 83.6% (online supplemental table S2). Voluntary feedback collected from parents who declined participation and clinicians’ views on factors affecting recruitment are summarised (online supplemental table S3).

10.1136/archdischild-2023-325871.supp5 Supplementary data

10.1136/archdischild-2023-325871.supp6 Supplementary data

#### Study completion and completeness of data collection

The proportion of randomised infants with complete data for the proposed primary outcome of catheter colonisation was 97/116 (83.6%) (online supplemental table S2). Completeness of data collection forms was excellent, with only two forms missing (from babies who did not complete the study) (online supplemental table S4).

10.1136/archdischild-2023-325871.supp7 Supplementary data

### Safety outcomes

One hundred and fourteen babies who received IMP underwent a total of 274 separate skin applications with allocated IMP (2% CHG-70% IPA, n=197; 2% CHG-aqueous n=77), comprising insertion and removal disinfections and applications that preceded failed catheterisation attempts (figure 1; online supplemental tables S2 and S5). Safety data were obtained for all 114 babies (100%) who received allocated antiseptic, including for babies transferred before catheter removal. Table 4 summarises daily skin morbidity scores in the period between catheter insertion and 48 hours post catheter removal (or post antiseptic application when catheterisation unsuccessful). No baby had any serious or major chemical burn injury or moderate/severe skin reaction recorded or requiring reporting after antiseptic application. A minority showed limited erythema (20/114; 17.5%); this appeared more common if catheterised in the first postnatal days and/or extremely preterm. Seven (6.1%) had limited skin breakdown/excoriation recorded (table 4). All skin morbidity was minor, self-limiting and resolved fully. None required special dressing or plastic surgical referral.

10.1136/archdischild-2023-325871.supp8 Supplementary data

**Table 4:** Daily skin morbidity scores in the period between catheter insertion and 48 hours post catheter removal

Skin morbidity scores	2% CHG-70% IPA (n=87)	2% CHG-aqueous (n=27)
Worst score for skin dryness throughout safety monitoring period		
Median (IQR)	1 (1–1)	1 (1–1)
Range	(1–2)	(1–2)
1	80 (92.0)	26 (96.3)
2	7 (8.0)	1 (3.7)
3	0	0
Worst score for erythema throughout safety monitoring period		
Median (IQR)	1 (1–1)	1 (1–1)
Range	(1–2)	(1–2)
1	72 (82.8)	22 (81.5)
2	15 (17.2)	5 (18.5)
3	0	0
Worst score for breakdown/excoriation throughout safety monitoring period		
Median (IQR)	1 (1–1)	1 (1–1)
Range	(1–2)	(1–2)
1	82 (94.3)	25 (92.6)
2	5 (5.7)	2 (7.4)
3	0	0
Worst score for totals of all three scores throughout safety monitoring period		
Median (IQR)	3 (3–4)	3 (3–4)
Range	(3–5)	(3–5)
3	65 (74.7)	20 (74.1)
4	20 (23.0)	6 (22.2)
5	2 (2.3)	1 (3.7)
≥6	0	0
Scoring was performed at baseline, within 10-30 minutes of catheterisation, and then daily until 48 hours post catheter removal, including for any infants repatriated to another hospital with their PCVC still in situ. Skin integrity scoring was also recorded until 48 hours post antiseptic application in instances where catheterisation proved unsuccessful. Skin scores were graded as follows:		
**Dryness** 1=Normal, no sign of dry skin 2=Dry skin, visible scaling 3=Very dry skin, cracking/fissures	**Erythema** 1=No evidence of erythema 2=Visible erythema <50% of skin area exposed to antiseptic 3=Visible erythema ≥50% of skin area exposed to antiseptic	**Breakdown/excoriation** 1=None evident 2=Small localised areas 3=Extensive

CHG, chlorhexidine gluconate; IPA, isopropyl alcohol; IQR, interquartile range; PCVC, percutaneous central venous catheter.

### Process outcomes

#### Catheterisation success rate

Catheterisation was successful in 106 (93%) of 114 babies who underwent attempted PCVC placement (figure 1). Online supplemental table S5 shows numbers of anatomical sites having at least one failed catheterisation.

#### Adherence to protocol

There was good adherence for the intervention (online supplemental table S5) and no major protocol breaches.

#### Withdrawals

There were no study infant withdrawals (figure 1).

## Discussion

We successfully carried out a feasibility RCT to compare alcohol versus aqueous formulations of 2% CHG. This is the first RCT to evaluate these formulations specifically for skin disinfection before PCVC insertion in preterm neonates. We have demonstrated a very low primary outcome incidence rate of only 4.1% of catheters being colonised with potentially pathogenic bacteria at the time of removal when 2% CHG-70% IPA antiseptic was used for skin disinfection prior to catheterisation. Furthermore, no major antiseptic-related skin injury was reported after application of either formulation under our strict working guideline. We completed recruitment within a 16-month period and had good rates of compliance with study procedures. Completeness of data collection was excellent, and we gathered rigorous prospective safety data for skin integrity. The primary and all planned secondary objectives were achieved. The ARCTIC trial demonstrates that it would in principle be feasible to conduct a definitive multicentre trial comparing the same two antiseptics in a non-inferiority study.

Our primary objective was to determine catheter colonisation rate in infants who received 2% CHG-70% IPA, to allow sample size calculation for a definitive efficacy study. Finding the catheter colonisation rate to be only 4.1% gave a much lower event rate than anticipated (~21%) at the outset.^3^ Modelling sample size for a definitive comparative non-inferiority study using the same primary outcome of catheter colonisation, detection of an absolute risk reduction of 2% would require ~n=3250 infants (90% power, two-sided significance level of 0.05). Assessing a composite clinical outcome of CRS+CAS instead: to detect an absolute risk reduction in catheter infections of 4% (from the combined incidence of CRS+CAS of 15% in our reference group down to 11%), we would need ~n=3400 (allowing for 10% loss-to-follow-up). For a non-inferiority hypothesis (to detect a non-inferiority margin of difference of no less than 4%), ~n=3700 would be needed (allowing for 10% loss-to-follow-up). So, while a definitive trial is feasible, these post hoc sample size calculations indicate that a very large trial would be needed.

The ~4% catheter colonisation rate seen in the ARCTIC trial reference group was much lower than the ~30% overall rate seen in our previous multicentre study that used much weaker strength (0.015% and 0.05%) CHG antiseptics.^1^ This sevenfold reduction is probably multifactorial: while the stronger CHG-plus-alcohol combined antiseptic trialled has likely played a major part, it is also likely that the rigorous methodology of catheter insertion and other good catheter care practices helped reduce catheter colonisation. We incorporated such practices into our study protocol to harmonise practices between sites and to maximise compliance with the elements of catheter care ‘bundles’ already collectively known to reduce catheter infection rates.^9 10^

The main limitation of our feasibility study is from the clinical perspective: the findings are inevitably limited for guiding current clinical practice for preferred antiseptic choice—for that requires a definitive large-scale RCT. Nevertheless, some trial findings may assist current practices. Our low outcome rate (~4%) of catheters colonised at removal after using 2% CHG-70% IPA antiseptic at catheterisation/pre-removal is a rigorous benchmark figure that other centres could reference to audit their own units’ rates of catheter colonisation using the same or other locally preferred antiseptic formulations. We encourage this and suggest that a national audit or registry may provide useful data. Also, our rigorous prospective safety data collected through daily skin monitoring provide preliminary reassurance that both these two ‘stronger’ 2% antiseptic formulations of CHG can be safely applied on the skin of preterm babies if used under similar carefully controlled guidelines (online supplemental files 1 and 2). We therefore propose that both agents merit inclusion in catheter care bundles for preterm babies. Our study adds to the existing but limited RCT evidence base for 2% CHG-70% IPA and 2% CHG-aqueous safety in preterm neonates.^24–26^ We nevertheless share cautions about their wider use in the first few days postnatal in the lowest gestation babies (<26 weeks) when the burden of skin colonisation is usually lightest yet the risk of chemical injury is greatest.^18^ It would therefore presently seem prudent to use lower concentration alcohol-free CHG preparations in the first few postnatal days, for example, 0.2% chlorhexidine acetate,^27^ although accepting the trade-off that rates of catheter sepsis may potentially then be higher.

### Conclusion and future study

The data from the ARCTIC study suggest that both 2% CHG-aqueous solution and 2% CHG-70% IPA can be used safely in preterm neonates when applied using a strict procedure to limit overexposure. Their use was associated with a large reduction in the risk of catheter colonisation by potentially harmful bacteria compared with historical rates using weaker preparations. A definitive trial is feasible, but based on the very low catheter colonisation rate or combined rate of CRS and CAS, a very large sample size is required. Newer agents such as octenidine^28^ now require formal evaluation in preterm neonates. But with such low rates of catheter colonisation and sepsis, conducting any definitive efficacy RCT of antiseptics now poses a formidable challenge. Other ways to distinguish between disinfection agents may be needed, such as registry or real-world data-based assessments of safety and efficacy, or else snapshot audits involving a limited number of centres willing to adopt uniform strict protocols and standardised procedures for catheter care and sampling.

## Data Availability

Data are available upon reasonable request. Reasonable requests for access to the data that support the findings of this study will be considered by contacting the corresponding author.
