# Supplementary material for: 2% chlorhexidine gluconate aqueous versus 2% chlorhexidine gluconate in 70% isopropyl alcohol for skin disinfection prior to percutaneous central venous catheterisation: the ARCTIC randomised controlled feasibility trial
Source: Arch Dis Child Fetal Neonatal Ed. 2023 Oct 31;109(2):202–10. doi: 10.1136/archdischild-2023-325871 (PMC10894828; doi:10.1136/archdischild-2023-325871)
Supplement: Supplementary data [file fetalneonatal-2023-325871supp001.pdf]

Affix Patient
ID Label Here

ARCTIC
Antiseptic Randomised Controlled Trial for Insertion of Catheters

Working Document 1

Study No:					
Pack No:					

STANDARDISED GUIDELINE FOR CATHETER INSERTION USING GOOD CATHETER INSERTION AND CARE PRACTICES

This procedure is to be performed with an assistant. Both the operator and assistant must be trained in good catheter insertion and care, and named on the ARCTIC study training or delegation log. The assistant's role is to monitor adherence to this working document. One document must be completed for EACH attempt.

For PCVC insertion equipment, see Appendix 1.

Each pack contains two bottles of the allocated antiseptic (identified by the same pack number) – as above.

Please read thoroughly and complete each point to ensure adherence to current protocol		Initial when done
1	Document number of this attempt (1,2,3,4...) ☐ Date of attempt: ___/___/___	
2	Check that the allocated antiseptic pack no. above corresponds with the pack no. on the bottle.	
3	Bottle No 1 / 2* from above Pack No. *Please circle<br>Date and time bottle opened ____/____/____ ____:____	
4	Document date and time opened on bottle used. NB. Each bottle of study antiseptic can be used for *up to 24 hours* after first being opened. If a second bottle is being used, a new pack will need to be allocated via the randomisation website for use when catheter is removed.	
5	Prescribe IMP on EPMA. (search "TRIAL" and you will find it listed in red as '**High Alert! TRIAL – ARCTIC STUDY Solution**')	
6	Place an ARCTIC IMP prescription label on the 'notice board' section of the baby's hard copy drug prescription chart.	
7	Use the dedicated percutaneous central venous catheter trolley, and ensure equipment from Appendix 1 is complete.	
8	Wash hands and clean trolley with Clinell wipe	
9	Following strict aseptic principles, open out the IV cut down set onto the cleaned trolley surface and add further equipment as required.	
10	Decant 3-5 mL only of the allocated solution into gallipot and ensure the IMP bottle is securely recapped	
11	Measure length of expected catheter insertion from selected insertion site(s) to intended location of catheter tip	
12	Document a baseline assessment of the skin where antiseptic is to be applied on chart on Appendix 2. (If there are any concerns about skin integrity, seek the advice of the research team or attendant consultant neonatologist prior to applying antiseptic)	
13	Apply face mask then wash hands up to elbows.	
14	Put on a sterile gown and double gloves, using strict aseptic non-touch technique.	
15	Prepare your equipment. (Handle the catheter with care, do not stretch or apply tension)	
16	Flush catheter with 0.9% saline and leave the syringe attached. **DO NOT** cut the catheter to alter the length.	
17	Assistant to damp dust the incubator ensuring the portholes are wiped with a Clinell wipe.	
18	Assistant to position the infant to facilitate insertion, ensuring that comfort measures and any pain medication is provided.	
19	With assistant's help, position the blue drape (minor ops pack) over the baby with the required insertion site available via the central aperture with the limb being held, as necessary, by your assistant to keep your field sterile.	

Clarke P, *et al. Arch Dis Child Fetal Neonatal Ed* 2024; 109:202–210. doi: 10.1136/archdischild-2023-325871

Norfolk and Norwich University Hospitals NHS Foundation Trust

Affix Patient
ID Label Here

ARCTIC
Antiseptic Randomised Controlled Trial for Insertion of Catheters

Working Document 1

Study No:					
Pack No:					

20	Soak gauze completely in allocated antiseptic and squeeze out thoroughly prior to application.	
21	Apply to the area selected for catheter insertion for a minimum of 10 seconds and maximum of 20 seconds. NB a **single** application of antiseptic is to be applied only. If catheterisation is done via a limb, the assistant should hold the limb through the aperture while the skin is disinfected by the operator. The operator can then fully take over the holding of the baby's limb using sterile gauze, holding the area already disinfected, before cleaning the remainder of the limb.<br>***NB Take great care to use only the minimal volume of antiseptic necessary for skin coverage, avoid any pooling of antiseptic, and ensure that any excess solution and any soaked materials, drapes, or gowns are removed to avoid any prolonged contact of antiseptic with the skin.***	
22	Allow the disinfected area to air dry completely (for at least 30 seconds) before proceeding with catheter insertion.<br>Do ***not*** use sterile water to wipe off the disinfected skin area after application of antiseptic solution (***unless*** catheter insertion has been ***unsuccessful)***, because this practice potentially negates the efficacy of the chlorhexidine antiseptic and will therefore potentially confound the study findings, and will constitute a violation of the protocol.	
23	Remove top pair of gloves and insert catheter aseptically as per Appendix 2.	
24	Following catheter insertion but prior to x-ray, assess skin integrity and document on chart on Appendix 2 (10-30 minutes post antiseptic application)	
25	Verify and document satisfactory catheter tip location via an x-ray.<br>If catheter position needs to be adjusted following x-ray, use strict aseptic technique when making any adjustments, and ensure a further check radiograph is obtained to document satisfactory position.	
**Is Catheter Insertion successful, confirmed by X-ray? Y / N**		
26	If **Y,** ensure the routine catheter insertion sticker is completed in baby's notes.<br><br>Time of Successful Catheter insertion ____:____<br><br>Type of PCVC inserted? (Please tick)<br><br>– Epicutaneo-Cava Catheter (24G) ☐<br><br>– Premicath (28G) ☐	
27	If **N**, Thoroughly clean with sterile water, the whole area that was subject to antiseptic application, and remove the catheter (If inserted) using standard practice.<br><br>Time of unsuccessful attempt ____:____	
28	Return all opened and unopened bottles of ARCTIC antiseptic to the IMP storage cupboard in Room 4.	
Note that the allocated IMP bottle may be used again within 24 hours of opening for subsequent catheterisation attempts in the same baby.		

Norfolk and Norwich University Hospitals NHS Foundation Trust

Affix Patient ID Label Here

ARCTIC
Antiseptic Randomised Controlled Trial for Insertion of Catheters

Working Document 1

Study No:					
Pack No:					

Confirmation of adherence

Please sign below to confirm that you have adhered to this Working Document.

	Operator	Assistant
Name:		
Job Title:		
Date:		
Signature:		

Appendix 1

Equipment

- Percutaneous central venous catheter trolley
- Clinell wipes for surface cleaning
- IV Cut down set
- Good source of light
- Minor ops pack
- Gown
- Mask
- 10 mL syringe
- 2 mL syringe
- Needleless connections (Bionectors)
- Tape measure
- Blunt needle (for drawing up the saline flush)
- 0.9% sodium chloride ampoule 10mls
- Sterile gauze – small and large
- Steristrips (Size 6 mm x 38 mm)
- Transparent sterile dressing
- Vygon: Epicutaneo-Cava Catheter 24G or Premicath 28G percutaneous central venous catheter
- Sterile gloves x2

Clarke P, *et al. Arch Dis Child Fetal Neonatal Ed* 2024; 109:202–210. doi: 10.1136/archdischild-2023-325871

Affix Patient
ID Label Here

ARCTIC
Antiseptic Randomised Controlled Trial for Insertion of Catheters

Working Document 1

Study No:					
Pack No:					

Appendix 2

Aseptic Catheter Insertion Technique

Apply tourniquet to limb (if necessary) using gauze, or have an assistant (who would then also need to be surgically gowned) apply pressure above the sterile site if necessary.<br>Anchor the vein by stretching the overlying skin with the thumb and fingers of the free hand.
Insert the green flagged needle/split needle or appropriate cannula through the skin about 0.5-1 cm distal to the intended vein at a low angle (15-30°)<br>When flash back occurs advance chosen cannula/needle appropriately.
Release the tourniquet (if used).<br>Introduce the primed catheter through the needle/cannula using non-toothed forceps and advance percutaneous central venous catheter to the desired length.
Secure the percutaneous central venous catheter in place using Steristrips.<br>If any dried blood needs to be removed from the skin following line insertion, sterile water may be used sparingly for this purpose prior to applying the transparent dressing, (i.e. do **not** use further IMP for this purpose)
When the area is completely dry, use the smallest amount of gauze possible and a transparent dressing to secure the PCVC in place, allowing the greatest area of the antiseptic site coverage to remain visible for skin observations. Aim to use a minimum number of Steristrips and the smallest necessary piece of gauze dressing.
Attach infusion of saline as standard practice at 0.5 mL/hr until line position is confirmed.

Appendix 3

Baseline Skin Assessment

**Region of which antiseptic to be applied.**	**Date and Time of baseline Skin Assessment** (before application of antiseptic)	**Dryness** *(tick one)*<br>1 = Normal, no sign of dry skin<br>2 = Dry skin, visible scaling<br>3 = Very dry skin, cracking/fissures	**Erythema** *(tick one)*<br>1 = No evidence of erythema<br>2 = Visible erythema <50% of skin area to be exposed to antiseptic<br>3 = Visible erythema ≥50% of skin area to be exposed to antiseptic	**Breakdown/excoriation** *(tick one)*<br>1 = None evident<br>2 = Small localised areas<br>3 = Extensive
	__/__/___ __:__			

Skin Assessment 10 – 30 minutes Post Antiseptic Application

**Region of which antiseptic has been applied.**	**Date and Time of post antiseptic Skin Assessment**	**Dryness** *(tick one)*<br>1 = Normal, no sign of dry skin<br>2 = Dry skin, visible scaling<br>3 = Very dry skin, cracking/fissures	**Erythema** *(tick one)*<br>1 = No evidence of erythema<br>2 = Visible erythema <50% of skin area to be exposed to antiseptic<br>3 = Visible erythema ≥50% of skin area to be exposed to antiseptic	**Breakdown/excoriation** *(tick one)*<br>1 = None evident<br>2 = Small localised areas<br>3 = Extensive
	__/__/___ __:__			

Clarke P, *et al. Arch Dis Child Fetal Neonatal Ed* 2024; 109:202–210. doi: 10.1136/archdischild-2023-325871

Affix Patient
ID Label Here

Working Document 1

Study No:					
Pack No:					

Clarke P, *et al. Arch Dis Child Fetal Neonatal Ed* 2024; 109:202–210. doi: 10.1136/archdischild-2023-325871
