# Supplementary material for: 2% chlorhexidine gluconate aqueous versus 2% chlorhexidine gluconate in 70% isopropyl alcohol for skin disinfection prior to percutaneous central venous catheterisation: the ARCTIC randomised controlled feasibility trial
Source: Arch Dis Child Fetal Neonatal Ed. 2023 Oct 31;109(2):202–10. doi: 10.1136/archdischild-2023-325871 (PMC10894828; doi:10.1136/archdischild-2023-325871)
Supplement: Supplementary data [file fetalneonatal-2023-325871supp002.pdf]

JENNY LIND CHILDREN'S HOSPITAL

Norfolk and Norwich University Hospitals NHS
NHS Foundation Trust

Affix patient ID
Label Here

ARCTIC
Antiseptic Randomised Controlled Trial for Insertion of Catheters

Study no: __ __ __ __ __

WORKING DOCUMENT 2

PCVC Removal Allocated Pack No: __ __ __ __ __

INSTRUCTIONS FOR CATHETER REMOVAL, OBTAINMENT OF STUDY SAMPLES AND SUBMISSION OF STUDY SPECIMENS TO LABORATORY -

Catheter removal will be carried out as a sterile procedure. An assistant will be needed to hold the baby still and remove the dressing.

Pre-prepared removal packs have been made up to facilitate catheter removal. These are stored in the clean utility room, on the top of row E/F – See appendix 1 for contents

Please read thoroughly and complete each point to ensure adherence to current protocol		Initial when done
1	Check that the allocated antiseptic pack number matches the pack number documented at the top of this page.	
2	Prescribe on EPMA, as previously done for line insertion. (Search "TRIAL" and you will find it listed in red as **'High Alert! TRIAL – ARCTIC STUDY Solution'**).	
3	Place an ARCTIC IMP prescription label on the 'notice board' section of the baby's hard copy drug prescription chart.	
4	Open the sterile pack onto the clean surface and empty a small amount of the allocated antiseptic solution into the gallipot.	
5	Disconnect catheter from fluid line, remove all external covering dressings of the PCVC and all Steristrips; inspect and record skin condition on the skin record form, Form 2 Section B.	
6	Document the catheter insertion length at the point of entry to the skin (see figure 1 overleaf)<br>**__ __.__cm**	
7	Wash hands, dry with sterile dressing towel and put on sterile gloves	
8	**Before** skin disinfection ***and*** **before** PCVC removal, take a first skin swab for microbial culture at the exact point of catheter insertion, covering no more than 0.5-1 cm diameter.	Time sample taken:
9	With the PCVC still in situ: using sterile gauze wetted with a minimal amount of *allocated* solution, squeeze out the gauze to remove excess antiseptic then carefully disinfect about a 1-2 cm diameter area of skin around the entry site of catheter insertion, for between 10 and 20 seconds. ***Ensure the exact puncture site is completely disinfected all around the catheter, including the exposed catheter, prior to removal.*** Take great care to ***avoid*** pooling of antiseptic.	
10	Leave the skin to dry for minimum of 30 seconds, ensuring the site is ***completely dry*** following the skin site disinfection	
11	***After skin site disinfection but before PCVC removal***, take a second skin swab for microbial culture at the ***exact*** point of the disinfected catheter insertion site, covering no more than 0.5-1 cm diameter.	Time sample taken:
12	Gently remove the catheter and place onto a sterile dressing towel, and document the date and time of line removal ___/___/___ ___:___	
13	Using one pair of sterile scissors and a pair of sterile forceps, cut the catheter tip segment (approx. 1 cm length) and place it into the universal sterile pot, labelled '**segment tip**'.	Time sample taken:
14	Using the **other** pair of sterile scissors and the **second** pair of forceps, obtain a segment of approximately 1 cm length by cutting at a distance 1–2 cm *inside* the former point of skin entry, (from the *previously-subcutaneous* portion of the catheter) and place this into the second sterile universal container, labelled '**proximal segment'**. (See figure 1 overleaf)	Time sample taken:
		**PTO**

Version 1.7 (NNUH) 06Dec2017
Working document 2

Norfolk and Norwich University Hospitals NHS Foundation Trust

Affix patient ID Label Here

ARCTIC Antiseptic Randomised Controlled Trial for Insertion of Catheters

Study no: __ __ __ __ __

WORKING DOCUMENT 2

PCVC Removal Allocated Pack No: __ __ __ __ __

15	Send all four specimens (catheter segments x2, skin swab x2) to microbiology using the ARCTIC drop down boxes on ICE, for routine culture and antibiotic sensitivities,ensuring that the bottles are labelled with ARCTIC study labels including study number, **as well as baby's own hospital bar code label**. *Please email laura.mansell@nnuh.nhs.uk to inform of specimens being sent and cc.in amy.nichols@nnuh.nhs.uk for her information.*	
16	If baby is considered clinically septic at time of catheter removal please ensure a blood culture is taken concurrently	

Figure 1.

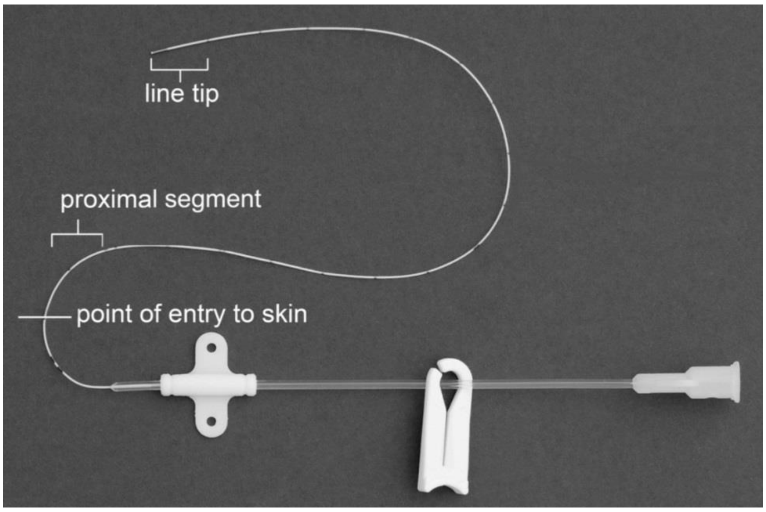

Continue to complete 'Neonatal Skin Condition Assessment' (Form 2, Section B) for 48 hours following catheter removal

N.B. All opened and unopened bottles of ARCTIC antiseptic must be replaced in the IMP cupboard in Room 4 for disposal by the Research Team.

Confirmation of adherence

The team of 'Catheter remover and Assistant' must please sign below to confirm adherence to this Working Document

	Person removing study catheter	Assistant
Name:		
Job Title:		
Date:		
Signature:		

Version 1.7 (NNUH) 06Dec2017
Working document 2

JENNY LIND CHILDREN'S HOSPITAL

Norfolk and Norwich University Hospitals NHS
NHS Foundation Trust

Affix patient ID
Label Here

ARCTIC
Antiseptic Randomised Controlled Trial
for Insertion of Catheters

WORKING DOCUMENT 2

Study no: __ __ __ __ __

PCVC Removal Allocated Pack No: __ __ __ __ __

Appendix 1

- **Catheter Removal Pack – Contents**
- 2 x sterile forceps
- 2 x sterile scissors
- 1 x ‘essential 1 wound care’ pack containing:
  - *1 dressing towel*
  - *1 gallipot*
  - *Sterile gauze*
- 2 x sterile universal pots, labelled ‘segment tip’ and ‘proximal segment’,
- 2 x charcoal skin swabs
- 1 x sterile dressing towel for drying hands

In addition you will require:

- 1 x pair of sterile gloves
- The **CORRECTLY** labelled ARCTIC antiseptic solution that was allocated to the baby at randomisation (stored in research IMP cupboard in assessment room)

Version 1.7 (NNUH) 06Dec2017
Working document 2
