# Supplementary material for: 2% chlorhexidine gluconate aqueous versus 2% chlorhexidine gluconate in 70% isopropyl alcohol for skin disinfection prior to percutaneous central venous catheterisation: the ARCTIC randomised controlled feasibility trial
Source: Arch Dis Child Fetal Neonatal Ed. 2023 Oct 31;109(2):202–10. doi: 10.1136/archdischild-2023-325871 (PMC10894828; doi:10.1136/archdischild-2023-325871)
Supplement: Supplementary data [file fetalneonatal-2023-325871supp003.pdf]

2

ARCTIC – Antiseptic Randomised Controlled Trial for Insertion of Catheters
Funded by NIHR Research for Patient Benefit Programme
ISRCTN: 82571474
eudraCT number: 2015-000874-36

UKCRC Registered Clinical Trials Units

NPEU Clinical Trials Unit

Supplemental material BMJ Publishing Group Limited (BMJ) disclaims all liability and responsibility arising from any reliance placed on this supplemental material which has been supplied by the author(s) Arch Dis Child Fetal Neonatal Ed

ARCTIC

Antiseptic Randomised Controlled Trial for Insertion of Catheters

The efficacy and safety of two topical antiseptic solutions for skin disinfection prior to percutaneous central venous catheter insertion in preterm neonates: a feasibility study

Statistical Analysis Plan
Version 1.0
Date: 8 August 2018

Author: *Christopher Partlett, Medical Statistician, NPEU CTU*
Reviewers: *Louise Linsell, Senior Medical Statistician, NPEU CTU*
Paul Clarke, Chief Investigator, Norfolk and Norwich University Hospital NICU
Ed Juszczak, Director, NPEU CTU
Kate Costeloe, Chair of TSC, Homerton University Hospital

Clarke P, *et al. Arch Dis Child Fetal Neonatal Ed* 2024; 109:202–210. doi: 10.1136/archdischild-2023-325871

Protocol version: v3.0

Supplemental material BMJ Publishing Group Limited (BMJ) disclaims all liability and responsibility arising from any reliance placed on this supplemental material which has been supplied by the author(s) Arch Dis Child Fetal Neonatal Ed

TABLE OF CONTENTS

1 Introduction 4
2 Trial personnel 4
3 Trial design and objectives 5
4 Description of outcomes and analysis populations 5
4.1 Primary outcome 5
4.2 Secondary outcomes 5
4.2.1 Feasibility measures 5
4.2.2 Efficacy measures 5
4.2.3 Safety measures 6
4.2.4 Process outcomes 6
5 Sample size and power 6
6 Random allocation 6
7 Protocol non-compliances 6
7.1 Major 7
7.2 Minor 7
7.2.1 Participants randomised in error 7
7.2.2 Participants who do not receive allocated intervention 7
8 Data collection schedule 7
9 Patient groups for analysis 8
9.1 Primary analysis strategy 8
9.2 Post-randomisation exclusions 8
9.3 Descriptive analysis population 8
9.4 Primary analysis population 8
9.5 Safety analysis population 8
10 Baseline characteristics 9
10.1 Representativeness of trial population and participant throughput 9
10.2 Baseline comparability of randomised groups 9
10.2.1 Neonatal characteristics 9
10.2.2 Maternal characteristics 9
10.3 Losses to follow-up 10
10.4 Adherence to intervention 10
11 Analysis of outcomes 11
11.1 Evaluation/definition of outcomes 11
11.2 Primary outcome 11

Clarke P, et al. Arch Dis Child Fetal Neonatal Ed 2024; 109:202–210. doi: 10.1136/archdischild-2023-325871

11.3 Secondary outcomes 11
11.3.1 Feasibility metrics 11
11.3.2 Bacterial species 11
11.3.3 Clinical outcomes 11
11.3.4 Completeness of data 12
11.3.5 Safety 12
11.4 Pre-specified subgroup analyses 12
11.5 Sensitivity analyses 12
Supplemental material BMJ Publishing Group Limited (BMJ) disclaims all liability and responsibility arising from any reliance placed on this supplemental material which has been supplied by the author(s) Arch Dis Child Fetal Neonatal Ed
11.6 Missing data 12
11.7 Statistical software employed 12
12 Safety data analysis 12
13 Additional exploratory analysis 12
14 References 13
14.1 Trial documents 13
14.2 Other references 13
15 Approval 13
16 Document history 13

Clarke P, *et al. Arch Dis Child Fetal Neonatal Ed* 2024; 109:202–210. doi: 10.1136/archdischild-2023-325871

BMJ Publishing Group Limited (BMJ) disclaims all liability and responsibility arising from any reliance placed on this supplemental material which has been supplied by the author(s)

1 INTRODUCTION

This document details the proposed presentation and analysis of the paper(s) reporting the two-centre randomised feasibility study ARCTIC, funded by the National Institute of Health Research (NIHR) – Research for Patient Benefit Programme.

The results reported in these papers will follow the strategy set out here. Subsequent analyses of a more exploratory nature will not be bound by this strategy, though they are expected to follow the broad principles laid down here. The principles are not intended to curtail exploratory analysis, nor to prohibit accepted practices, but they are intended to establish the rules that will be followed, as closely as possible, when analysing and reporting the trial.

The analysis plan will be available on request when the principal papers are submitted for publication in a journal. Suggestions for subsequent analyses by journal editors or referees, will be considered carefully, and carried out as far as possible in line with the principles of this analysis plan; if reported, the source of the suggestion will be acknowledged.

Any deviations from the statistical analysis plan will be described and justified in the final report of the trial. The analysis should be carried out by an identified, appropriately qualified and experienced statistician, who should ensure the integrity of the data during their processing. Examples of such procedures include quality control and evaluation procedures.

2 TRIAL PERSONNEL

Chief Investigator
Dr Paul Clarke
Consultant Neonatologist
Norfolk and Norwich University Hospitals NHS Foundation Trust
paul.clarke@nnuh.nhs.uk

Trial Coordinator
Tracy Oliver
Norfolk and Norwich University Hospitals NHS Foundation Trust
tracy.oliver@nnuh.nhs.uk

Trial Statisticians
Jennifer Bell (since June 2018), Christopher Partlett (previous) and Louise Linsell
NPEU Clinical Trials Unit, University of Oxford
jennifer.bell@npeu.ox.ac.uk, christopher.partlett@npeu.ox.ac.uk, louise.linsell@npeu.ox.ac.uk

CTU Director
Associate Professor Ed Juszczak
NPEU Clinical Trials Unit, University of Oxford
ed.juszczak@npeu.ox.ac.uk

Supplemental material BMJ Publishing Group Limited (BMJ) disclaims all liability and responsibility arising from any reliance placed on this supplemental material which has been supplied by the author(s) Arch Dis Child Fetal Neonatal Ed

Trial Programmers
Andy King and David Murray
NPEU Clinical Trials Unit, University of Oxford
andy.king@npeu.ox.ac.uk, david.murray@npeu.ox.ac.uk

3 TRIAL DESIGN AND OBJECTIVES

ARCTIC is a two-centre blinded randomised feasibility study of two topical antiseptics for neonatal skin disinfection prior to insertion of a percutaneous central venous catheter (PCVC) in the neonatal intensive care unit.

Preterm infants born at <34 weeks' gestation who are undergoing planned insertion of a PCVC will be randomised to receive one of two commonly used topical disinfection agents for skin antisepsis: aqueous-based 2% chlorhexidine gluconate (2%CHG), or 70% isopropyl alcohol-based 2% chlorhexidine gluconate (70%IPA/2%CHG).

The primary objective of this feasibility study is to estimate the prevalence of central venous catheter bacterial colonisation at the time of catheter removal in the 70%IPA/2%CHG arm, in order to inform the sample size calculation for a phase-III trial.

4 DESCRIPTION OF OUTCOMES AND ANALYSIS POPULATIONS

4.1 Primary outcome

Proportion of babies in the 70%IPA/2%CHG arm with catheter colonisation as determined by positive bacterial culture from at least one of the two catheter segments taken at catheter removal.

4.2 Secondary outcomes

4.2.1 Feasibility measures

- Rates of recruitment and retention to the study, and the collection of views of parents and clinicians on factors affecting recruitment and retention
- Proportion of infants completing study with complete data for the primary outcome
- Proportions of infants with missing data collection forms.

4.2.2 Efficacy measures

- Proportion of infants with positive exit-site skin swabs (ESSS) at catheter removal (before and after skin disinfection)
- Number and type of catheter segments culture positive at removal
- Bacterial species (typed via molecular methods) of isolates identified on positive BC, ESSS (before and after skin disinfection), and catheter segment
- Proportion of infants undergoing an infection screen in the period between catheter insertion and 48 hours post-catheter removal that meets case definition for definite catheter-related sepsis.

Clarke P, *et al. Arch Dis Child Fetal Neonatal Ed* 2024; 109:202–210. doi: 10.1136/archdischild-2023-325871

- Proportion of infants with positive blood culture from any infection screen in the period between catheter insertion and 48 hours post-catheter removal that meets definition for catheter-associated sepsis
- Rate of catheter-related sepsis per 1000 PCVC days
- Rate of catheter-associated sepsis per 1000 PCVC days.

4.2.3 Safety measures

Supplemental material BMJ Publishing Group Limited (BMJ) disclaims all liability and responsibility arising from any reliance placed on this supplemental material which has been supplied by the author(s) Arch Dis Child Fetal Neonatal Ed

- Daily skin morbidity scores in the period between catheter insertion and 48 hours post-catheter removal, and in the period between antiseptic application and 48 hours post-antiseptic application where catheterisation was unsuccessful.

4.2.4 Process outcomes

- Number of anatomical sites at which a PCVC insertion was attempted and failed
- Adherence to study protocol
- Study withdrawals.

5 SAMPLE SIZE AND POWER

ARCTIC is using a 3:1 allocation ratio in favour of the reference 70%IPA/2%CHG group. A target sample size of approximately 93 babies with successfully inserted catheters (approximately n=70 in the reference group) will be necessary to estimate the critical parameters for a future, large-scale trial with the desired degree of precision. If this target sample size is achieved in the feasibility study, the anticipated incidence of the primary outcome (catheter colonisation) in the reference group of 20% will be estimated with a 95% confidence interval (CI) of 11% to 31%.

With a sample size of 93 babies with successfully inserted catheters, the anticipated recruitment/uptake rate of 75% will be estimated with a 95% CI of 65% to 83%. To obtain a sample size in the region of 93 babies having catheters successfully inserted will require parents of at least 124 eligible babies to be consented. Based on our previous collaborative observational study of PCVCs that recruited 127 preterm infants between two tertiary centres in a 14-month study period, we would expect to complete recruitment within 14 months.

6 RANDOM ALLOCATION

Randomisation is carried out using permuted block randomisation with variable block sizes and stratifying on birth gestation (<28 weeks; 28^{+0} to 33^{+6} weeks) and neonatal centre. The randomisation will use a 3:1 allocation ratio in favour of allocating to the alcohol-based antiseptic (70%IPA/2%CHG).

Clarke P, et al. Arch Dis Child Fetal Neonatal Ed 2024; 109:202–210. doi: 10.1136/archdischild-2023-325871

7 PROTOCOL NON-COMPLIANCES

All protocol non-compliances will be listed in the final report. Non-compliances are defined below.

BMJ Publishing Group Limited (BMJ) disclaims all liability and responsibility arising from any reliance
placed on this supplemental material which has been supplied by the author(s)

7.1 Major

The following are pre-defined major protocol non-compliances with a direct bearing on the primary outcome:

- Data considered fraudulent
- Infants randomised without informed maternal consent.

7.2 Minor

7.2.1 Participants randomised in error

These included infants who did not meet the eligibility criteria:

- Born at greater than or equal to 34 weeks' gestation
- Have an underlying skin condition
- Already have an indwelling PCVC in situ or was previously enrolled in respect of an earlier PCVC episode
- Have a positive blood culture in the 7 days prior to randomisation without a subsequent negative blood culture result
- Have had antibiotic treatment for suspected sepsis within the 48 hours preceding randomisation.

7.2.2 Participants who do not receive allocated intervention

- Infants randomised to receive aqueous-based 2% chlorhexidine gluconate (2%CHG) but instead receive alcohol-based (70% isopropyl alcohol) 2% chlorhexidine gluconate (70%IPA/2%CHG)
- Infants randomised to receive alcohol-based (70% isopropyl alcohol) 2% chlorhexidine gluconate (70%IPA/2%CHG) but instead receive aqueous-based 2% chlorhexidine gluconate (2%CHG).
- Infants randomised to either intervention who do not receive either allocated intervention.

8 DATA COLLECTION SCHEDULE

Information will be collected using the following study-specific data collection forms:

- Form 1: Trial Entry and Randomisation Form
- Outcome Data Collection Forms
    - Form 2: Main Outcome Data Form
    - Form 3: Unsuccessful Catheterisation Episode Form
    - Form 4: PCVC Removal Form
    - Form 5: Microbiology Data Form
- Form 6: Discontinuation of Intervention Form
- Form 7: Withdrawal Form
- Form 8: End of Study
- Form 9: Foreseeable Serious Adverse Event Form.

In addition, information will be collected and reported to the Sponsor using the Sponsor's reporting forms, as follows:

- Form 10: Serious Adverse Event (SAE/SUSAR) report Form
- Incident Form (Form for Protocol Deviation, Violation, Breach or Serious Breach of Protocol or GCP) .

9 PATIENT GROUPS FOR ANALYSIS

BMJ Publishing Group Limited (BMJ) disclaims all liability and responsibility arising from any reliance placed on this supplemental material which has been supplied by the author(s)

9.1 Primary analysis strategy

Where possible, infants will be analysed in the groups to which they are randomly assigned, regardless of deviation from the protocol or treatment received (referred to as the Intention to Treat (ITT) population).

However, most outcome measures (including the primary outcome) require a catheter to be successfully inserted. For these outcomes the analysis will be carried out on a 'modified ITT' population: infants with successfully inserted catheters will be analysed in the groups to which they were randomised.

Since ARCTIC is a feasibility study the analysis will be predominantly descriptive. For instance, the primary outcome only relates to infants randomised to a single arm. However, some of the outcomes (i.e. the clinical outcomes listed in section 11.3.3) will be analysed by arm and comparative results will be presented.

9.2 Post-randomisation exclusions

After randomisation, in the following circumstances infants will be excluded from the analysis population(s):

(i) major protocol non-compliance
(ii) infants for whom consent to use their data has been withdrawn
(iii) infants that did not receive either intervention because no study catheter insertion attempt was ever made for them

9.3 Descriptive analysis population

Baseline neonatal and maternal characteristics will be reported for all infants randomised for whom we have data available, excluding post-randomisation exclusions.

9.4 Primary analysis population

All infants randomised, excluding post-randomisation exclusions.

Clarke P, et al. Arch Dis Child Fetal Neonatal Ed 2024; 109:202–210. doi: 10.1136/archdischild-2023-325871

9.5 Safety analysis population

All infants randomised that received at least one application of one of the study antiseptics, including infants where catheterisation was unsuccessful.

10 BASELINE CHARACTERISTICS

10.1 Representativeness of trial population and participant throughput

The flow of participants through each stage of the trial will be summarised using a CONSORT diagram. We will report the numbers of infants:

- Assessed for eligibility (screened)
- Not eligible
- Eligible
- Could not be recruited because parents declined
- Missed recruitment for other reasons (e.g. staff unavailable)

Supplemental material BMJ Publishing Group Limited (BMJ) disclaims all liability and responsibility arising from any reliance placed on this supplemental material which has been supplied by the author(s) Arch Dis Child Fetal Neonatal Ed

- Randomised
- Allocated to each intervention
- Did not receive allocated intervention
- Post-randomisation exclusions
- Randomised in error (e.g. duplicate randomisation)
- Withdrawn consent
- Discontinued intervention
- Successfully inserted catheter
- Included in the analysis of safety outcomes
- Included in the analysis of primary outcome.

10.2 Baseline comparability of randomised groups

Participants in the original two randomised groups will be described separately with respect to maternal and infant characteristics at trial entry:

10.2.1 Neonatal characteristics

- Centre
- Sex
- Birthweight
- Gestational age
- Multiple pregnancy
- Mode of delivery
- Membranes ruptured prior to labour
- Membranes ruptured >24 hours before delivery
- Apgar score at 5 minutes
- First recorded temperature on admission to the neonatal unit after birth
- Infant ventilated via an endotracheal tube at the time of randomisation
- Infant born in this hospital
- Any surgical procedure prior to randomisation

Clarke P, et al. Arch Dis Child Fetal Neonatal Ed 2024; 109:202–210. doi: 10.1136/archdischild-2023-325871

- Prophylactic antifungal medication at the time of randomisation
- Infant received antibiotics prior to randomisation
- Devices in situ at time of PCVC insertion.

10.2.2 Maternal characteristics

- Age (years)

- Any antenatal corticosteroids
- Antibiotics within the week before delivery
- Pyrexial in labour (temperature >38.0 °C)
- Chorioamnionitis suspected clinically before delivery.

Supplemental material BMJ Publishing Group Limited (BMJ) disclaims all liability and responsibility arising from any reliance placed on this supplemental material which has been supplied by the author(s) Arch Dis Child Fetal Neonatal Ed

Numbers (with percentages) for binary and categorical variables and means (with standard deviations), or medians (with lower and upper quartiles, and minimum and maximum) for continuous variables will be presented. There will be no tests of statistical significance performed nor confidence intervals calculated for differences between randomised groups on any baseline characteristic.

10.3 Losses to follow-up

The number (with percentages) of losses to follow-up among infants will be reported for the two trial arms, and the reasons will be recorded. This is likely to be minimal, as follow-up ends 48 hours after catheter removal (successful insertion and removal of study catheter) or 48 hours after last IMP application (for unsuccessful catheterisation). Any deaths (and their cause) will be reported separately.

There is anticipated to be some loss to follow-up caused by infants with successful or unsuccessful catheter insertions transferring to non-participating sites. For these infants, safety data will be sought up to 48 hours after catheter removal (successful insertion and removal of study catheter) or 48 hours after last IMP application (for unsuccessful catheterisation). For infants lost to recruiting study site with study catheter indwelling, attempts will be made to gather data relevant to important clinical secondary outcome measures, including whether blood culture was done as part of an infection screen while the study catheter remained indwelling or in the 48-hour period following its removal within the non-participating neonatal unit providing ongoing clinical care.

Where possible, the catheter tip will be returned to the recruiting site for microbiological analysis. While these results will not contribute to the primary outcome, they will be useful for a secondary analysis (section 13).

10.4 Adherence to intervention

Adherence to the intervention will be assessed using the following questions from the Main Outcome Data Form:

- **QA3:** Was the insertion done observing strict aseptic technique and in accordance with Working Document "Standardised guideline for catheter insertion utilising good catheter insertion and care practices"?
- **QA7:** Was the insertion site disinfected with the allocated study antiseptic prior to successful PCVC insertion?
- **QA8:** Confirm that baseline skin condition is recorded to describe the PCVC insertion site appearance prior to successful PCVC insertion?
- **QA9:** Was the allocated study antiseptic used to clean the skin before PCVC insertion applied sparingly and for 10 to 20 seconds?

Clarke P, et al. Arch Dis Child Fetal Neonatal Ed 2024; 109:202–210. doi: 10.1136/archdischild-2023-325871

- **QA10:** Was the allocated study antiseptic allowed to dry for at least 30 seconds prior to the successful PCVC insertion?
- **QA11:** Following skin disinfection preceding the successful PCVC insertion, can you confirm that no other solution was used to wipe off the antiseptic from the skin?

and using the following question from the PCVC Removal Form:

- **QA5:** exit site disinfected after first skin swab taken but before PCVC removal?

Adherence to the protocol can also be assessed from the number of deviation forms.

Supplemental material
BMJ Publishing Group Limited (BMJ) disclaims all liability and responsibility arising from any reliance placed on this supplemental material which has been supplied by the author(s)
Arch Dis Child Fetal Neonatal Ed

11 ANALYSIS OF OUTCOMES

11.1 Evaluation/definition of outcomes

The primary outcome will be analysed and reported for infants in the 70%IPA/2%CHG arm only. Rates of recruitment will be analysed and presented for both arms combined, while retention will be summarised by arm and overall. The proportion of infants with missing data collection forms will be summarised for both arms combined. All other secondary outcomes (including process outcomes) will be analysed and presented by arm.

Derivation of variables is described in the data derivation spreadsheet.

11.2 Primary outcome

The primary outcome is the proportion of babies in the 70%IPA/2%CHG arm with catheter colonisation as determined by positive bacterial culture from at least one of the two catheter segments taken at catheter removal.

The proportion of babies with catheter colonisation will be reported along with a 95% confidence interval.

11.3 Secondary outcomes

11.3.1 Feasibility metrics

The following key feasibility metrics will be reported:

- Uptake rate — proportion of eligible infants who are randomised
- Retention — proportion of infants randomised who remain in the study to provide primary outcome data and complete safety data.

These will be reported as proportions with 95% confidence intervals. In addition, the views of parents and clinicians on factors affecting recruitment and retention will be collected and reported.

11.3.2 Bacterial species

The bacterial species (typed via molecular methods) of isolates identified on positive blood culture (BC), ESSS (before and after skin disinfection), and catheter segment will be listed by arm for infants with a positive blood culture.

Clarke P, *et al. Arch Dis Child Fetal Neonatal Ed* 2024; 109:202–210. doi: 10.1136/archdischild-2023-325871

11.3.3 Clinical outcomes

The following clinical outcomes will be reported as proportions (or rates) in each arm:

Supplemental material BMJ Publishing Group Limited (BMJ) disclaims all liability and responsibility arising from any reliance placed on this supplemental material which has been supplied by the author(s) Arch Dis Child Fetal Neonatal Ed

- Proportion of infants with positive exit-site skin swabs (ESSS) at catheter removal (before and after skin disinfection)
- Number and type of catheter segments culture positive at removal
- Proportion of infants undergoing an infection screen in the period between catheter insertion and 48 hours post-catheter removal that meets case definition for definite catheter-related sepsis
- Proportion of infants with positive blood culture from any infection screen in the period between catheter insertion and 48 hours post-catheter removal that meets definition for catheter-associated sepsis
- Rate of catheter-related sepsis per 1000 PCVC days
- Rate of catheter-associated sepsis per 1000 PCVC days.

11.3.4 Completeness of data

The extent of missing data will be reported for every outcome. The number and percentage of missing forms will be reported for all infants combined for each form.

11.3.5 Safety

Daily skin morbidity scores will be summarised by arm for all infants, including those where catheterisation was unsuccessful. These will be compared between arms using either a difference in means or a difference in medians, along with a 95% confidence interval.

11.4 Pre-specified subgroup analyses

None planned.

11.5 Sensitivity analyses

None planned.

11.6 Missing data

Missing data will be described by presenting the number of individuals in the missing category. As the sample size is small, imputation techniques will not be appropriate.

11.7 Statistical software employed

Stata/SE 13.1 or later for Windows.

12 SAFETY DATA ANALYSIS

Clarke P, et al. Arch Dis Child Fetal Neonatal Ed 2024; 109:202–210. doi: 10.1136/archdischild-2023-325871

Unforeseeable serious adverse events (SAEs) and suspected unexpected serious adverse reactions (SUSARs) will be listed by trial allocation.

13 ADDITIONAL EXPLORATORY ANALYSIS

A secondary exploratory analysis of the primary outcome, catheter colonisation, will be carried out to assess the sensitivity of primary outcome if the definition changed to include only proximal line segment alone, or tip segment alone.

14 REFERENCES

14.1 Trial documents

Protocol ARCTIC_protocol_v3.0 dated 18th November 2016
ARCTIC Data Derivation
ARCTIC Dummy Tables

14.2 Other references

None yet.

Supplemental material BMJ Publishing Group Limited (BMJ) disclaims all liability and responsibility arising from any reliance placed on this supplemental material which has been supplied by the author(s) *Arch Dis Child Fetal Neonatal Ed*

15 APPROVAL

Senior Statistician	Name: LOUISE LINSELL		
	Signature [redacted]	Date 08/08/18.	
Chief Investigator	Name: PAUL CLARKE		
	Signature [redacted]	Date 08/AUG/2018	
Chair of Trial Steering Committee (or delegate)	Name: KATE COSTELOE		
	Signature [redacted]	Date 24/08/18	

16 DOCUMENT HISTORY

Version	Date	Edited by	Comments/Justification	Timing in relation to interim analysis/unblinding
0.1	20/03/17	CP	Initial draft created	Prior to both
0.2	01/06/17	CP	Changes made following input from PC: Analysis populations for the outcomes clarified & derivation of variables updated.	Prior to both
0.3	05/06/17	CP	Updated following review by LL.	Prior to both
0.4	13/07/17	CP	Updated following outcome mappings meeting with PC.	Prior to both

Clarke P, *et al. Arch Dis Child Fetal Neonatal Ed* 2024; 109:202–210. doi: 10.1136/archdischild-2023-325871

Supplemental material
BMJ Publishing Group Limited (BMJ) disclaims all liability and responsibility arising from any reliance placed on this supplemental material which has been supplied by the author(s)
Arch Dis Child Fetal Neonatal Ed

			Included details on adherence measures.<br><br>Added comments relating to changes to be made regarding sample size in version 4.0 of protocol.	
0.5	23/08/17	CP	Minor edits following review by LL.	Prior to both
0.6	13/11/17	CP	Revisions following review by EJ	Prior to both
0.7	15/12/17	CP	Revised following review by PC. Clarified which randomised infants will be excluded from the analysis. Added detail to ESSS (before and after disinfection)	Prior to both
0.8	16/01/18	CP	Revised following further review by PC. Added two extra baseline characteristics. Added details of an exploratory secondary analysis of the primary outcome.	Prior to both
0.9	08/03/18	CP	Revised following DMC meeting on 26th January 2018. Removed RR column from secondary outcomes table. Added details of safety analysis population to CONSORT.	After unblinding and first interim analysis
0.10	06/04/18	CP	Revised following review by TSC. Clarified description of baseline characteristics. Created a new subgroup of outcomes: feasibility measures.	After unblinding and first interim analysis
0.11	21/06/18	CP	Revisions approved by PC. Minor changes to wording. Minor rewording of primary outcome for clarity.	After unblinding and first interim analysis
1.0	08/08/18	JB	Amended section 12 Safety Data Analysis, so SAEs and SUSARs will be listed by allocation instead. Final version for sign-off.	After unblinding and first interim analysis

Clarke P, et al. *Arch Dis Child Fetal Neonatal Ed* 2024; 109:202–210. doi: 10.1136/archdischild-2023-325871
