# Supplementary material for: 2% chlorhexidine gluconate aqueous versus 2% chlorhexidine gluconate in 70% isopropyl alcohol for skin disinfection prior to percutaneous central venous catheterisation: the ARCTIC randomised controlled feasibility trial
Source: Arch Dis Child Fetal Neonatal Ed. 2023 Oct 31;109(2):202–10. doi: 10.1136/archdischild-2023-325871 (PMC10894828; doi:10.1136/archdischild-2023-325871)
Supplement: Supplementary data [file fetalneonatal-2023-325871supp004.pdf]

Supplementary Table S1: Supplementary information related to catheterisation

	70%IPA-2%CHG (n = 79)	2%CHG aqueous (n = 27)	Overall (n = 106)
Postnatal age (days) at line insertion, median (IQR)	5.0 (2.0 to 7.0)	4.0 (2.0 to 8.0)	5.0 (2.0 to 7.0)
Range (min to max)	(0 to 46)	(1 to 19)	(0 to 46)
Catheter inserted in ≤ 3 days after birth, n (%)	31 (39.2)	11 (40.7)	42 (39.6)
Anatomical site of long line insertion			
Upper limb (inc. axilla)	46 (58.2)	15 (55.6)	61 (57.5)
Lower limb	32 (40.5)	11 (40.7)	43 (40.6)
Scalp	1 (1.3)	1 (3.7)	2 (1.9)
At least one blood culture sent while line was in situ, n (%)	25 (33.3)	8 (32.0)	33 (33.0)
Missing	4	2	6
Time (days) to first positive blood culture during line indwell in infants that had bloodstream infection, N	11	4	15
Median (IQR)	7.0 (5.0 to 10.0)	5.5 (5.0 to 9.5)	6.0 (5.0 to 10.0)
**Line removal details completed, N**	**78**	**26**	**104**
Postnatal age (days) at line removal, median (IQR)	13.0 (10.0 to 19.0)	15.0 (11.0 to 21.0)	14.0 (10.0 to 20.0)
Range (min to max)	(3 to 57)	(3 to 29)	(3 to 57)
Duration of line indwelling, median (IQR)	9.0 (6.0 to 12.0)	9.5 (7.0 to 12.0)	9.0 (6.0 to 12.0)
Range (min to max)	(1 to 32)	(2 to 20)	(1 to 32)
Reason for removal, n (%) (non-exclusive)			
No longer needed	68 (87.2)	20 (76.9)	88 (84.6)
Suspected sepsis	5 (6.4)	3 (11.5)	8 (7.7)
Confirmed sepsis	3 (3.8)	0 (0.0)	3 (2.9)
Damaged	0 (0.0)	1 (3.8)	1 (1.0)
Unintended removal	1 (1.3)	0 (0.0)	1 (1.0)
Blocked	2 (2.6)	1 (3.8)	3 (2.9)
Malposition confirmed by x-ray	4 (5.1)	1 (3.8)	5 (4.8)
Other complications	1 (1.3)	1 (3.8)	2 (1.9)
Missing	1	1	2
Blood culture sent at time of PCVC removal in those with suspected or confirmed sepsis, N	8	3	11
n (%)	5 (62.5)	3 (100.0)	8 (72.7)
Antibiotics received on day of PCVC removal, n (%)	15 (19.2)	6 (23.1)	21 (20.2)
At least one dose prior to removal, n (%)	11 (78.6)	4 (66.7)	15 (75.0)
Missing	1	0	1
Within 7 days before removal, n (%)	10 (100.0)	4 (100.0)	14 (100.0)
Missing	1	0	1
