# Supplementary material for: 2% chlorhexidine gluconate aqueous versus 2% chlorhexidine gluconate in 70% isopropyl alcohol for skin disinfection prior to percutaneous central venous catheterisation: the ARCTIC randomised controlled feasibility trial
Source: Arch Dis Child Fetal Neonatal Ed. 2023 Oct 31;109(2):202–10. doi: 10.1136/archdischild-2023-325871 (PMC10894828; doi:10.1136/archdischild-2023-325871)
Supplement: Supplementary data [file fetalneonatal-2023-325871supp005.pdf]

Supplementary Table S2: Rates of recruitment and retention

	**Total eligible[1] (n = 178)**
**Uptake rate**	
Number of eligible infants randomised, n (%)	116 (65.2)
Proportion (95% CI)	65.2 (57.7, 72.1)

	**70%IPA-2%CHG (n = 88)**	**2%CHG aqueous (n = 28)**	**All (n = 116)**
**Retention[2]**			
Number of infants who remained in the study, n (%)	73 (83.0)	24 (85.7)	97 (83.6)
Proportion (95% CI)	83.0 (73.4, 90.1)	85.7 (67.3, 96.0)	85.1 (77.2, 91.1)

[1]Eligible infants were defined as those who were recruited or not recruited (including those whose parents declined their participation) but who were clinically eligible.
[2]Proportion of infants that remained in the study to provide complete primary outcome and safety data. The overall proportion of randomised infants with complete data for the proposed primary outcome of catheter colonisation was 97/116 (83.6%). Considering only babies who had successfully inserted catheters, 97/106 (91.5%) had both proximal and tip catheter segment cultures available for analysis.
