# Supplementary material for: 2% chlorhexidine gluconate aqueous versus 2% chlorhexidine gluconate in 70% isopropyl alcohol for skin disinfection prior to percutaneous central venous catheterisation: the ARCTIC randomised controlled feasibility trial
Source: Arch Dis Child Fetal Neonatal Ed. 2023 Oct 31;109(2):202–10. doi: 10.1136/archdischild-2023-325871 (PMC10894828; doi:10.1136/archdischild-2023-325871)
Supplement: Supplementary data [file fetalneonatal-2023-325871supp006.pdf]

Supplementary Table S3: Parents' and clinicians' views on factors affecting recruitment

Main reasons volunteered by parents for declining consent, n
Not interested in participating in any research, 2
Already enrolled in another study and did not want to join another, 2
Concern about skin reaction to the antiseptic, 3
Felt their baby had been very sick, did not want to impose anything else on them, 3
Parents of twins who did not want one in a study without the other being enrolled, 1
Parents of twins who did not want to participate because they wanted to ensure their babies received the unit's standard alcohol-based 2% chlorhexidine antiseptic for catheterisation as they considered this would be superior, 1
No reason offered, 21
**Main reasons provided by clinical staff for factors affecting recruitment**
Time pressure – too busy with clinical work to be able to approach/consent
Parents not available to discuss participation
Urgent central venous access needed (eg umbilical venous catheter insertion had been unsuccessful in the first hours after birth so urgent PCVL needed)
New personnel, not yet trained in study procedures
Not GCP certified so unable to obtain consent
Eligibility for the study was overlooked

T
