# Supplementary material for: 2% chlorhexidine gluconate aqueous versus 2% chlorhexidine gluconate in 70% isopropyl alcohol for skin disinfection prior to percutaneous central venous catheterisation: the ARCTIC randomised controlled feasibility trial
Source: Arch Dis Child Fetal Neonatal Ed. 2023 Oct 31;109(2):202–10. doi: 10.1136/archdischild-2023-325871 (PMC10894828; doi:10.1136/archdischild-2023-325871)
Supplement: Supplementary data [file fetalneonatal-2023-325871supp007.pdf]

Supplementary Table S4: Completeness of data collection

	**Total[1] (n = 106)**
Infants with no missing data collection forms, n (%)	104 (98.1)
Overall form completeness for required forms, n (%)	
Form 1: Trial Entry and Randomisation Form	106 (100.0)
Form 2: Main Outcome Data Form	106 (100.0)
Form 4: PCVC Removal Form	104 (98.1)[2]
Form 5: Microbiology Data Form	106 (100.0)
Form 8: End of Study	106 (100.0)

[1] Analysed for clinical outcomes – infants who had a successfully inserted catheter and received the intervention
[2] Two participants did not complete the study as they were transferred to non participating hospitals before study catheter removal, and so PCVC Removal Forms were not required.
