# Supplementary material for: 2% chlorhexidine gluconate aqueous versus 2% chlorhexidine gluconate in 70% isopropyl alcohol for skin disinfection prior to percutaneous central venous catheterisation: the ARCTIC randomised controlled feasibility trial
Source: Arch Dis Child Fetal Neonatal Ed. 2023 Oct 31;109(2):202–10. doi: 10.1136/archdischild-2023-325871 (PMC10894828; doi:10.1136/archdischild-2023-325871)
Supplement: Supplementary data [file fetalneonatal-2023-325871supp008.pdf]

Supplementary Table S5: Process outcomes and adherence to protocol

	70%IPA-2%CHG (n = 87)	2%CHG aqueous (n = 27)
Number of anatomical sites with at least one failed PCVC insertion		
Median (IQR)	0 (0 to 1)	1 (0 to 2)
Range	(0 to 7)	(0 to 4)
1	18 (20.7)	6 (22.2)
2	10 (11.5)	6 (22.2)
3	1 (1.1)	0
4	1 (1.1)	2 (7.4)
**Adherence to intervention**		
Successful catheterisation (N)	79	27
Insertion done observing strict aseptic technique and in accordance with Working Document “Standardised guideline for catheter insertion utilising good catheter insertion and care practices", n (%)	79 (100.0)	27 (100.0)
Insertion site disinfected with the allocated study antiseptic prior to successful PCVC insertion, n (%)	79 (100.0)	27 (100.0)
Baseline skin condition is recorded to describe the PCVC insertion site appearance prior to successful PCVC insertion, n (%)	79 (100.0)	27 (100.0)
Allocated study antiseptic used to clean the skin before PCVC insertion applied sparingly and for 10 to 20 seconds, n (%)	79 (100.0)	27 (100.0)
Allocated study antiseptic allowed to dry for at least 30 seconds prior to the successful PCVC insertion, n (%)	79 (100.0)	27 (100.0)
Following skin disinfection preceding the successful PCVC insertion, no other solution was used to wipe off the antiseptic from the skin[1], n (%)	76 (96.2)	26 (96.3)
Exit site disinfected after first skin swab taken but before PCVC removal[2], n (%)	75 (96.2)	24 (92.3)
Missing	1	1

[1] Two infants in the 70%IPA/2%CHG arm and one in the 2%CHG arm who did have another solution had sterile water used.

[2] Three infants in the 70%IPA/2%CHG arm and one in the 2%CHG arm who didn’t have their exit site disinfected at this time had their line removed at a non-participating site. For one infant in the 2%CHG arm who didn’t, the allocated solution could not be located.
